# Low BALF CD4 T cells count is associated with extubation failure and mortality in critically ill covid-19 pneumonia

**DOI:** 10.1080/07853890.2022.2095012

**Published:** 2022-07-04

**Authors:** Gurmeet Singh, Cleopas Martin Rumende, Surendra K. Sharma, Iris Rengganis, Zulkifli Amin, Tonny Loho, Emmy Hermiyanti, Kuntjoro Harimurti, Heri Wibowo

**Affiliations:** a Department of Internal Medicine, Faculty of Medicine, Division of Respirology and Critical Illness, Universitas Indonesia – Cipto Mangunkusumo General Hospital, Jakarta, Indonesia; b Department of Molecular Medicine, Jamia Hamdard Institute of Molecular Medicine, Hamdard University, New Delhi, India; c Department of General Medicine & Pulmonary Medicine, JNMC, Datta Meghe Institute of Medical Science, New Delhi, India; d Department of Internal Medicine, Faculty of Medicine, Division of Allergy and Clinical Immunology, Universitas Indonesia, Cipto Mangunkusumo General Hospital, Jakarta, Indonesia; e Department of Clinical Pathology, Faculty of Medicine, Universitas Indonesia – Cipto Mangunkusumo General Hospital, Jakarta, Indonesia; f Department of Internal Medicine, Faculty of Medicine, Division of Respirology and Critical Illness, Universitas Padjadjaran, Dr Hasan Sadikin Hospital Bandung, Bandung, Indonesia; g Department of Internal Medicine, Faculty of Medicine, Division of Geriatrics, Universitas Indonesia – Cipto Mangunkusumo General Hospital, Jakarta, Indonesia; h Head of Integrated Laboratory, Faculty of Medicine, Universitas Indonesia, Jakarta, Indonesia

**Keywords:** Critically ill COVID-19 pneumonia, BALF CD4 T-cells count, extubation failure

## Abstract

**Background:**

Critically ill COVID-19 pneumonia is one of the main causes of extubation failure and mortality. Understanding clinical characteristics, laboratory profiles and bronchoalveolar lavage fluid (BALF) immunopathology may help improve outcomes in critically ill COVID-19 pneumonia. We aimed to describe clinical characteristics, laboratory profiles and BALF immunopathology based on lung severity in critically ill COVID-19 pneumonia patients.

**Materials and methods:**

Forty critically ill severe pneumonia patients requiring invasive mechanical ventilation in Cipto Mangunkusumo General (National Tertiary Referral Hospital), Indonesia within November 2020–January 2021 were enrolled in this study. Early BALF collection was performed after patients’ intubation. Clinical characteristics, laboratory profiles and BALF biomarkers (sTREM-1, alveolar macrophage amount and function, IL-6, IL-17, CD4 T-cells, Tregs, SP-A and Caspase-3) were observed and analysed. Outcomes were measured based on extubation failure (within 19 days) and 28-days mortality. Univariate and bivariate analyses were performed.

**Results:**

Early bronchoscopy was performed in an average of 4 h (SD = 0.82) after patients’ intubation. Twenty-three and twenty-two patients had extubation failure (within 19 days) and 28-days mortality, respectively. In the baseline clinical characteristics of critically ill COVID-19 patients, we found no significant differences in the extubation and mortality status groups. In the laboratory profiles of critically ill COVID-19 patients, we found no significant differences in the extubation status groups. In critically ill COVID-19 pneumonia patients, there was a significant high D-dimer levels in survived group (*p* = .027), a significant low BALF CD4 T-cells count in the right lung (*p* = .001) and a significant low BALF CD4 T-cells count (*p* = .010 and *p* = .018) in severely affected lung with extubation failure and mortality.

**Conclusions:**

BALF CD4 T-cells count evaluation of severely affected lung is associated with early extubation failure and mortality in critically ill COVID-19 pneumonia patients.

**KEY MESSAGE**
- Few studies have been conducted during the peak COVID-19 period analysing combined bronchoalveolar lavage fluid (BALF) immunopathology biomarkers within four hours of intubation to assess extubation failure and mortality. In this study, we reported eight BALF immunopathology biomarkers (sTREM-1, alveolar macrophage, IL-6, IL-17, CD4 T-cells, Tregs, SP-A and Caspase-3).
- We found significantly low BALF CD4 T-cells count in the right lung, and low BALF CD4 T-cells count in severely affected lung of critically ill COVID-19 pneumonia patients in extubation failure and mortality.

## Introduction

Pneumonia involves persistent local inflammatory reactions which potentially increase disease severity due to the inability to control microbial invasion. Comprehensive knowledge regarding local inflammation may help understand the local host response to microorganisms [1]. The number of viral pneumonia cases increased during the coronavirus disease 2019 (COVID-19) pandemic [2]. Alteration of immune response (loss of immune control) in COVID-19 can prevent viral elimination and recovery [3]. A significant number of patients with critically ill COVID-19 pneumonia experienced severe respiratory failure, necessitating admission to the intensive care unit (ICU), with almost 80% of them requiring mechanical ventilation [4]. Due to various organ involvement and dynamic development over time, these patients exhibit high complexity. Various studies have described comorbidities, clinical scoring systems and laboratory profiles to determine the disease severity, ICU admission, the need for mechanical ventilation, and mortality [4–10]. However, little information is known concerning local immunopathology responses with the increased usage of the mechanical ventilator. Understanding clinical characteristics and bronchoalveolar lavage fluid (BALF) immunopathology in the severely and less severely affected lung may help to improve outcomes in critically ill COVID-19 pneumonia patients [11]. Additionally, the study of local inflammatory biomarkers of the lung is required to measure the severity of lung injury and determine the risk for extubation failure and mortality [12]. Previous studies have been conducted to analyse local immunopathology responses in sputum and bronchoalveolar lavage (BAL) fluid observing inflammatory profiles (i.e. cytokine interleukin (IL)-6, IL-8, IL-10, IL-17, IL-1β, IL-1ra, tumour necrosis factor-α (TNF-α) and interferon-γ (IFN-γ) [1,13], moreover, no study has been done to evaluate BALF immunopathology biomarker (sTREM-1, alveolar macrophage, IL-6, IL-17, CD4 T-cells, Tregs, SP-A and Caspase-3) based on the severity in the right and left lung of critically ill COVID-19 pneumonia patients. Each of these immunopathology biomarkers (sTREM-1 [14–18], alveolar macrophage [19–22], IL-6 [23–25], IL-17 [26–29], CD4 T-cells [30–32], Tregs [33–35], SP-A [36,37] or Caspase-3 [38,39]) has been studied independently to mediate local inflammatory responses in severe pneumonia patients. Based on human and animal BALF studies, we assumed that BALF sTREM-1 [18], alveolar macrophage [20], IL-6 [13], IL-17 [28], CD4 T-cells and Tregs [35], SP-A [40], and Caspase-3 [37] played a crucial role in local immunopathology (innate and adaptive immune responses) in critically ill COVID-19 pneumonia patients. Therefore, this study may give a better knowledge to understand wider perspective about BALF immunopathology biomarkers to determine outcomes in critically ill COVID-19 pneumonia patients.

The main aim of this study is to describe BALF immunopathology based on severity in right and left lung of critically ill COVID-19 pneumonia patients on mechanical ventilation hospitalized in Resuscitation Emergency Unit/Intensive Care Unit (REU/ICU). We analysed BALF biomarkers which may be associated with extubation failure (within 19 days) and 28-days mortality.

## Materials and methods

Forty critically ill severe pneumonia patients were enrolled in Cipto Mangunkusumo General (National Tertiary Referral Hospital), Indonesia within November 2020 to January 2021. The main teaching hospital for Universitas Indonesia is Cipto Mangunkusumo General Hospital (National Tertiary Referral Hospital). Inclusion criteria: aged 18 years or older; severe pneumonia (based on IDSA/ATS 2007 criteria and COVID-19 WHO guidelines confirmed by positive BALF PCR SARS-CoV-2); can undergo bronchoscopy within 12 h of admission to REU/ICU; receive empirical antibiotics of no more than 24 h; and intubated within 24 h. Exclusion criteria: acute respiratory distress syndrome (ARDS) non-infection; HIV/AIDS (confirmed by rapid anti-HIV testing); active malignancy within the last 12 months; on immunosuppressant therapy; refused to undergo bronchoscopy. Clinical characteristics, laboratory profiles and eight BALF biomarkers (sTREM-1, alveolar macrophage (amount and function), IL-6, IL-17, CD4 T-cells, Tregs, SP-A and Caspase-3) were recorded and analysed. This study has been approved by Ethics Committee Universitas Indonesia (approval number: KET-171/UN2.F1/ETIK/PPM.00.02/2020) and Ethics Committee of Health Research, Cipto Mangunkusumo Hospital. Informed consent was approved by ethics committee (approval number: 0995/rev00/KEP/2020). Informed consent for bronchoscopy was signed by patient’s family member (patients were intubated).

### Study design

The present study was designed as an observational cross-sectional study, in which eligible patients were classified into extubation (success and failure) group and mortality (survived and non-survived) group. Critically ill COVID-19 pneumonia patients will be further analysed to evaluate BALF immunopathology.

### Definition of extubation failure and mortality

The 19-days extubation failure was selected based on the study reported by Gamberini et al. (median 20-days extubation failure) [6], and 28-days mortality observation was chosen according to the reported pneumonia studies on mortality prediction [5,9]. Extubation failure was defined as patients being reintubated and/or reusing ventilator after successful extubation in 48 h, and/or death within 19 days [6,41]. Twenty-eight days mortality was defined as patients being recorded died in REU/ICU. Patients’ mortality outside REU/ICU (i.e. inward, after a hospital discharged) was not recorded as 28-days mortality.

### Assessment of lung severity

Radiographic chest X-ray findings were reported by radiologists prior to BALF collection. Severely and less severely affected lung were discussed by two respirologist and critical illness consultants, plus one internist, based on the affected lung chest imaging severity scoring proposed by Feng et al. [42].

### Bronchoalveolar lavage fluid collection

Early BALF collection was performed after patients’ intubation. Intravenous midazolam and propofol were given by the anaesthesiologist in order to give optimum sedation during bronchoscopy. The order of BAL suctioning was initially performed from the less severely affected lung and proceeded to the severely affected lung, from the subsegment of the right middle lobe and lingula of the left lung. Severely and less severely affected lung BALF were analysed separately. Bronchoalveolar lavage was performed (standard guidance [43]) by serial 20 mL fractions 0.9% of normal saline solution to a total volume of 100 mL (room temperature). Minimum of 60–70% of lavage volume was retrieved by gentle syringe suction collected to the mucus extractor in a wedge position and processed for further examination within 2 h. Patients were observed for one-hour post-procedure.

### Bronchoalveolar lavage fluid preparation

The BALF specimen containers were inserted in sterilized medical plastic bag prior to transfer to the Integrated Laboratory of medical faculty, Universitas Indonesia. Specimen containers were gathered in a ventilated room. All specimen handling was coordinated by experienced laboratory staff with a sufficient protective equipment including protective, disposable aprons, N95 masks, goggles and double layer hand gloves. Specimen volume was evaluated for appearance, colour, clearness and contamination with intrabronchial blood. Bronchoalveolar lavage fluid specimens were collected to 50 mL tube and then centrifuged at 1000×*g* for 10 min. Bronchoalveolar lavage fluid supernatant was separated to analyse sTREM-1, IL-6, IL-17 and SP-A and frozen at −80 °C. Bronchoalveolar lavage fluid pellet was suspended in 2 mL PBS (phosphate buffer saline) to analyse alveolar macrophage, Tregs and Caspase-3. For Caspase-3, sample was frozen at −80 °C prior to transfer to Integrated Laboratory of Medical Faculty, Universitas Indonesia.

### Bronchoalveolar lavage fluid analysis

Flow cytometry was used to analyse alveolar macrophage, CD4 T-cells and Tregs. Enzyme-linked immunosorbent assay (ELISA) was used in duplicate to analyse sTREM-1 (MyBioSource ELISA kits, San Diego, CA), IL-6 and IL-17 (R&D systems quantikine ELISA kits, Minneapolis, MN), SP-A (LSBio’s ELISA kits, Seattle, WA) and Caspase-3 (Cusabio ELISA kits, Houston, TX). Interleukin-6, IL-17 and SP-A were observed from BALF supernatant. Caspase-3 was observed from cell pellet homogenate and were extracted by using freeze-thawing method (this process was done for two times). Since BALF protein level is in the same concentration as in blood, total protein levels were used as an index of BALF dilution [44]. Bradford technique (Bio-RAD, Hercules, CA) was used to measure normalized protein in BALF supernatant or cell pellet.

### Flow cytometry BALF analysis

The obtaining cell pellet (alveolar macrophage and Tregs) was incubated at room temperature in the dark, with monoclonal antibodies (mAbs) in 5 mL polystyrene tubes for 15 min, followed by insertion of FACS lysing solution for 15 min. The working panel of mAbs at five colour assays used for alveolar macrophage and Tregs BALF evaluation was the following: anti-human CD206 PE, anti-human HLA-DR FITC, anti-human CD11b APC, anti-human CD45 PerCP-Cy5.5 and anti-human CD169 BV421 (BD Biosciences, Franklin Lakes, NJ). The resulting cell pellet of CD4 T-cells was incubated at room temperature in the dark, with mAbs in BD TruCount™ Tubes for 15 min, followed by insertion of FACS lysing solution for 15 min at room temperature with following mAbs working panel: CD45+/CD3+/CD4+/CD8+ (BD Biosciences, Franklin Lakes, NJ). Specimens were acquired with a FACSCanto™ II flow cytometer (BD Biosciences, Franklin Lakes, NJ) after been washed. BD FASCDiva™ software version 6.1.3 (BD Biosciences, Franklin Lakes, NJ) was used to perform the cytometric analysis.

Amount and function of alveolar macrophage were analysed according to procedure proposed by Nayak et al. [45] Alveolar macrophage (amount) was analysed according to percentage macrophage cells/CD45^+^, HLA-DR^+^ and CD11b^+^. Alveolar macrophage (function) was analysed according to the mean fluorescence intensity (MFI) of CD169^+^.

### Statistical analysis

Numerical and categorical variables were reported through mean, and standard deviation or median, and interquartile range (IQR), and proportions. Data normality was assessed based on Shapiro–Wilk’s test. A *t*-test was used to compare normal distribution variables (parametric data), and a Mann–Whitney Wilcoxon test was used to compare non-normal distribution continuous variables (non-parametric data). A Chi-square test was used for comparing categorical variables. Univariate analysis was performed for subject characteristics (demographic, clinical variables and laboratory values) and bivariate analysis was performed for biomarker variables (sTREM-1, alveolar macrophage, IL-6, IL-17, CD4 T-cells, Tregs, SP-A and Caspase-3). All *p* values <.05 were considered statistically significant. SPSS Statistic version 26 software (IBM Corporation, Armonk, NY) was used to analyse all recorded data.

## Results

Based on our national guidelines, all critically ill COVID-19 pneumonia patients in this study received intravenous dexamethasone, N-acetyl cysteine, low dose vitamin D supplementation, empirical antibiotics; and four patients received tocilizumab. Bronchoalveolar lavage fluid collection was performed in an average of 4 h (SD = 0.82) after patients’ intubation. Twenty-three and twenty-two patients had extubation failure and mortality, respectively.

Among 40 severe pneumonia patients, 28 critically ill COVID-19 pneumonia patients were further analysed to evaluate BALF immunopathology. Twelve patients were excluded (non-COVID-19) from this study. The baseline clinical characteristics of critically ill COVID-19 patients based on extubation and mortality status are shown in Tables 1 and 2, respectively. In the extubation status, we found no significant differences between group of extubation success and extubation failure of critically ill COVID-19 patients, as reported in Table 1.

**Table 1.** Clinical characteristics of critically ill COVID-19 patients on extubation status.

Variable	Extubation success (*n* = 5)	Extubation failure (*n* = 23)	*p*
Age, years	64 (±8)	61 (±11.5)	.550
Gender (male, *n*, %)	3 (60)	13 (56)	1
BMI (kg/m^2^), mean (±SD)	26.53 (±4)	26.85 (±3.42)	.855
Smoker, *n* (%)	0 (0)	7 (30)	.154
Severely affected lung (right, *n*, %)	3 (60)	16 (70)	.678
Comorbidities			
Diabetes mellitus, *n* (%)	3 (60)	13 (56)	.887
Hypertension, *n* (%)	3 (60)	11 (48)	.662
Chronic kidney disease, *n* (%)	1 (20)	9 (39)	.418
Scoring system			
APACHE II score, mean (±SD)	14.20 (±3.70)	16.30 (±5.15)	.397
mSOFA score, median (IQR)	8.00 (8.00–8.00)	9.00 (8.00–10.00)	.099
CCI, mean (±SD)	5.20 (±2.68)	4.57 (±2.10)	.565

SD: standard deviation; IQR: interquartile range; APACHE: acute physiology and chronic health evaluation; mSOFA: modified sequential organ failure assessment; CCI: comorbidity channel index.

**Table 2.** Clinical characteristics of critically ill COVID-19 patients on mortality status.

Variable	Survived (*n* = 6)	Non-survived (*n* = 22)	*p*
Age, years	63 (±8)	61 (±12)	.746
Gender (male, *n*, %)	3 (50)	13 (59)	1
BMI (kg/m^2^), mean (±SD)	27.57 (±4.4)	26.58 (±3.24)	.542
Smoker, *n* (%)	0 (0)	7 (32)	.111
Severely affected lung (right, *n*, %)	4 (66)	15 (68)	.944
Comorbidities			
Diabetes mellitus, *n* (%)	4 (66)	12 (54)	.595
Hypertension, *n* (%)	4 (66)	10 (50)	.357
Chronic kidney disease, *n* (%)	1 (17)	9 (41)	.272
Scoring system			
APACHE II score, mean (±SD)	13.50 (±3.73)	16.60 (±5.07)	.178
mSOFA score, median (IQR)	8.00 (8.00–8.00)	9.00 (8.00–10.00)	.052
CCI, mean (±SD)	5.00 (±2.45)	4.59 (±2.15)	.691

SD: standard deviation; IQR: interquartile range; APACHE: acute physiology and chronic health evaluation; mSOFA: modified sequential organ failure assessment; CCI: comorbidity channel index.

In the mortality status, we found no significant differences between group of survived and non-survived of critically ill COVID-19 patients, as reported in Table 2.

Radiographic findings were reported by Radiologists in Cipto Mangunkusumo General (National Tertiary Referral Hospital). Chest X-ray findings in critically ill COVID-19 patients predominantly showed bilateral consolidation (*n* = 27) with severely affected right lung (*n* = 19), as shown in Appendix 3. The laboratory profiles of critically ill COVID-19 patients based on extubation and mortality status are shown in Tables 3 and 4, respectively. In the extubation status, we found no significant differences between group of extubation success and extubation failure of critically ill COVID-19 patients, as reported in Table 3.

**Table 3.** Laboratory profiles of critically ill COVID-19 patients on extubation status.

Lab profiles	Extubation success (*n* = 5)	Extubation failure (*n* = 23)	*p*
Haemoglobin (g/dL), mean (±SD)	14.16 (±1.20)	13.84 (±2.83)	.808
Leukocytes (×10^3^ cells/μL), mean (±SD)	15.15 (±4.82)	12.80 (±5.05)	.352
Neutrophils (%), mean (±SD)	85.66 (±4.60)	84.21 (±7.27)	.676
Lymphocytes (%), median (IQR)	11.30 (6.70–12.00)	9.90 (5.05–13.45)	.928
CRP (mg/L), mean (±SD)	191.38 (±155.83)	172.30 (±116.14)	.756
Ferritin (ng/mL), median (IQR)	2647.60 (1479.67–3510.22)	2333.00 (1259.55–3312.57)	.787
Albumin (g/dL), mean (±SD)	2.87 (±0.35)	3.18 (±0.49)	.194
D-dimer (μg/L), median (IQR)	9560 (6890–18,160)	1880 (1385–8670)	.063
PaO_2_/FiO_2_ ratio, median (IQR)	93.40 (47.60–95.54)	55.11 (48.65–92.74)	.653
Absolute CD4+ (cells/μL), mean (±SD)	229.60 (±132.851)	204.73 (±114.485)	.673
Serum IL-6 (pg/mL), median (IQR)	47.11 (32.65–48.14)	49.47 (27.30–89.84)	.529
Serum IL-17 (pg/mL), mean (±SD)	11.55 (±1.12)	11.88 (±0.78)	.756

CRP: C-reactive protein; PaO_2_/FiO_2_: the ratio of arterial oxygen partial pressure to fractional inspired oxygen; CD: cluster of differentiation; IL: interleukin.

**Table 4.** Laboratory profiles of critically ill COVID-19 patients on mortality status.

Lab profiles	Survived (*n* = 6)	Non-survived (*n* = 22)	*p*
Haemoglobin (g/dL), mean (±SD)	14.45 (±1.28)	13.74 (±2.86)	.566
Leukocytes (×10^3^ cells/μL), mean (±SD)	14.50 (±4.59)	12.87 (±5.16)	.489
Neutrophils (%), mean (±SD)	84.21 (±5.43)	84.54 (±7.26)	.920
Lymphocytes (%), median (IQR)	11.65 (6.70–13.60)	9.45 (5.00–13.00)	.654
CRP (mg/L), median (IQR)	182.00 (109.20–291.00)	140.15 (81.80–250.00)	.758
Ferritin (ng/mL), mean (±SD)	2459.72 (±1970.76)	2636.88 (±1853.21)	.893
Albumin (g/dL), mean (±SD)	2.82 (±0.33)	3.21 (±0.48)	.076
D-dimer (μg/L), median (IQR)	12,645 (6890–18,160)	1800 (1370–7400)	**.027**
PaO_2_/FiO_2_ ratio, median (IQR)	70.50 (40.00–95.54)	56.55 (49.00–103.75)	.780
Absolute CD4+ (cells/μL), mean (±SD)	266.33 (±149.049)	193.05 (±103.009)	.176
Serum IL-6 (pg/mL), median (IQR)	47.62 (32.65–67.04)	45.68 (27.16–88.90)	.955
Serum IL-17 (pg/mL), mean (±SD)	11.67 (±1.05)	11.87 (±0.80)	.628

CRP: C-reactive protein; PaO_2_/FiO_2_: the ratio of arterial oxygen partial pressure to fractional inspired oxygen; CD: cluster of differentiation; IL: interleukin.

In the mortality status, we found a significant difference of D-dimer between group of survived and non-survived of critically ill COVID-19 patients (12,646 vs. 1800 μg/L; *p* = .027), as reported in Table 4.

In the analysis of severely and less severely affected right and left lung, there were significantly lower BALF CD4 T-cells count in the right lung of critically ill COVID-19 patients (14 vs. 28 cells/μL; *p* = .001), as reported in Table 5.

**Table 5.** BALF immunopathology biomarkers in right and left lung.

Biomarkers	Right lung (*n* = 28)	Left lung (*n* = 28)	*p*
sTREM (ng/mg protein), median (IQR)	1.23 (0.89–2.88)	1.16 (0.82–2.46)	.179
Alveolar macrophage			
% (Macrophage cells/CD45+, HLA-DR+, CD11b+), median (IQR)	7.30 (2.75–10.20)	6.00 (2.45–10.05)	.909
MFI CD169, median (IQR)	701 (347.50–858.50)	610 (347–948.50)	.855
IL-6 (pg/mg protein), median (IQR)	332.02 (153.29–612.24)	270.41 (124.28–514.67)	.187
IL-17 (pg/mg protein), median (IQR)	7.56 (5.32–9.77)	5.68 (4.54–9.29)	.072
CD4 T-cells (cells/μL), median (IQR)	14 (8.5–24)	28 (15.5–53.50)	**.001**
Foxp3+ CD25+/CD4 (%), median (IQR)	19.64 (13.09–30.67)	14.38 (10.93–25.95)	.524
Caspase 3 (ng/mg protein), median (IQR)	0.82 (0.44–1.44)	0.70 (0.40–1.27)	.305
SP-A (ng/mg protein), median (IQR)	15.26 (9.32–19.48)	13.07 (10.97–14.66)	.194

### Bronchoalveolar lavage fluid immunopathology in severely affected lung of critically ill COVID-19 pneumonia with extubation success and failure

Bronchoalveolar lavage fluid immunopathology profiles are shown in Tables 6 and 7. In the group of extubation failure, we found significantly lower BALF CD4 T-cells count (56 vs. 11 cells/μL; *p* = .010), as reported in Table 6.

**Table 6.** BALF immunopathology biomarkers of severely affected lung on extubation status.

Biomarkers	Extubation success (*n* = 5)	Extubation failure (*n* = 23)	*p*
sTREM (ng/mg protein), median (IQR)	0.92 (0.3–3.71)	1.23 (0.35–10.72)	.453
Alveolar macrophage			
% (Macrophage cells/CD45+, HLA-DR+, CD11b+), mean (±SD)	8.4 (±8.44)	6.98 (±4.23)	.584
MFI CD169, median (IQR)	672 (308–738)	775 (217–7765)	.384
IL-6 (pg/mg protein), median (IQR)	173.52 (60.27–378.63)	337.65 (34.7–1689.95)	.197
IL-17 (pg/mg protein), median (IQR)	6.11 (3.83–10.05)	6.3 (1.86–29.31)	.976
CD4 T-cells (cells/μL), median (IQR)	56 (16–102)	11 (4–70)	**.010**
Foxp3+ CD25+/CD4 (%), median (IQR)	21.38 (6.71–28.13)	16.35 (4.5–81.4)	.976
Caspase 3 (ng/mg protein), median (IQR)	0.99 (0.56–2.54)	0.82 (0.14–5.33)	.384
SP-A (ng/mg protein), mean (±SD)	12.79 (±3.62)	14.86 (±5.43)	.427

MFI: mean fluorescence intensity.

**Table 7.** BALF immunopathology biomarkers of severely affected lung on mortality status.

Biomarkers	Survivors (*n* = 6)	Non-survivors (*n* = 22)	*p*
sTREM (ng/mg protein), median (IQR)	1.26 (0.3–3.71)	1.17 (0.37–10.72)	.780
Alveolar macrophage			
% (Macrophage cells/CD45+, HLA-DR+, CD11b+), mean (±SD)	8.7 (±7.59)	6.8 (±4.28)	.443
MFI CD169, median (IQR)	512.5 (308–738)	775 (217–7765)	.218
IL-6 (pg/mg protein), median (IQR)	218.25 (60.27–378.63)	375.51 (34.7–1689.95)	.218
IL-17 (pg/mg protein), median (IQR)	7.8 (3.83–29.31)	6.1 (1.86–27.17)	.467
CD4 T-cells (cells/μL), median (IQR)	40 (14–102)	11 (4–70)	**.018**
Foxp3+ CD25+/CD4 (%), median (IQR)	17.8 (6.71–28.13)	16.93 (4.5–81.4)	.654
Caspase 3 (ng/mg protein), median (IQR)	1.4 (0.56–5.33)	0.77 (0.14–2.91)	.117
SP-A (ng/mg protein), mean (±SD)	13.76 (±4.0)	14.7 (±5.5)	.701

MFI: mean fluorescence intensity.

### Bronchoalveolar lavage fluid immunopathology in severely affected lung of survivors and non-survivors critically ill COVID-19 pneumonia

In the group of non-survivors, we found significantly lower BALF CD4 T-cells count (40 vs. 11 cells/μL; *p*=.018), as reported in Table 7.

Bronchoalveolar lavage fluid immunopathology biomarkers in less severely affected lung of critically ill COVID-19 are reported in Appendixes 1 and 2.

## Discussion

The role of biomarkers has been suggested to guide clinicians for ventilator management in determining the potential risk of extubation failure and mortality [12]. This study implicates that the role of BALF CD4 T-cells as a biomarker is reliable for early management prediction in critically ill COVID-19 pneumonia patients (i.e. ventilator management, early tracheostomy and the need for aggressive treatment) which could shorten the length of hospital stays and to determine the readiness for extubation in patients who are at high risk for extubation failure.

Based on sample size calculation, 1010 patients were required in this study. Due to pandemic beta variant COVID-19 in Indonesia (November 2020 to January 2021), after the enrolment of 40 patients, an internal discussion was held, and the decision was to discontinue patients’ enrolment. To our knowledge, only a few studies were conducted during the peak COVID-19 period analysing BALF immunopathology to assess extubation failure and mortality [46]. Additionally, this is the first study to perform early bronchoscopy after patients’ intubation (average of 4 h, SD = 0.82) in severely and less severely affected lung to enumerate BALF immunopathology. Most BALF studies during pandemic COVID-19 have analysed cellularity characteristics [47–50] (i.e. CD4 T-cells, CD8 T-cells, macrophages). However, no data are available for combined BALF immunopathology biomarkers (i.e. sTREM, IL-6, IL-17, SP-A and Caspase-3).

Based on extubation and mortality status, we also observed clinical characteristics and laboratory profiles of critically ill COVID-19 pneumonia patients. D-dimer levels were significantly higher in the group of survived rather than non-survived. The role of elevated D-dimer in critically ill COVID-19 has been associated with disease severity and mortality [51]. Other studies have found elevated D-dimer to be associated with extubation failure and death [52,53]. Our study found a different result for elevated D-dimer probably due to small sample size, no serial D-dimer evaluation, and selection bias.

Several studies reported bilateral lung consolidation as frequent abnormalities findings in COVID-19 patients [54,55]. In this study, the majority of patients’ chest radiographs were characterized by bilateral diffuse consolidation, with the right lung being the most severely affected. Differences in anatomical structure led to a higher likelihood of foreign particles and pathogens entering the lower respiratory tract of the right lung [56–58]. Hence, the severity of inflammation will predominate in the right lung (marked by significantly low BALF CD4 T-cells count), as shown in this study. In the severe inflammation process, CD4 T-cells will differentiate into several subsets, including Tregs [59]. This study also displayed a high level of Tregs in extubation success and survived groups, albeit statistically not significant. This result indicates the vital function of Tregs as an immune suppressor in helping the adaptive immune process [60], and its role in the lung epithelial regeneration and surfactant production [61], as well as the potential effect on the resolution of the lung [62]. Future studies are required to investigate the detailed mechanisms of Tregs and the association with extubation success or extubation failure, which may better explain this present study.

Limited data are available for assessing extubation failure in critically ill COVID-19 pneumonia patients [7]. Information focussing on extubation failure in severe pneumonia patients has been reported by Yu et al. [10] Critically ill COVID-19 pneumonia patients need more prolonged intubation time [63], and Ionescu et al. [7] reported extubation failure in critically ill COVID-19 pneumonia was strongly associated with hospital mortality. Length of stays, requirement of tracheostomy and mortality were associated with extubation failure [64].

A BALF study by Saris et al. found that critically COVID-19 pneumonia patients showed a reduced levels of BALF CD4 T-cells count in non-survivor compared to the survivor group [46]. This study reports a significant low levels of BALF CD4 T-cells count in severely affected lung of critically ill COVID-19 pneumonia patients in extubation failure and non-survived group.

It is recommended for clinicians to evaluate BALF in severe pneumonia patients [65]. However, limited BALF immunopathology studies were found in severe pneumonia patients. Meta-analysis by Zeng et al. [66] reported serum immunopathology in critically ill COVID-19 pneumonia is associated with disease severity and high mortality. Sputum and BALF inflammatory biomarkers, IL-6, IL-8, IL-10, IL-17, IL-1β, IL-1ra, TNF-α and IFN-γ, have been reported in several studies [1,13]. Bronchoalveolar lavage fluid immunopathology biomarkers have been described to predict clinical outcomes in COVID-19 pneumonia patients [67]. Eight BALF immunopathology biomarkers were assessed in this study, sTREM-1, alveolar macrophage, IL-6, IL-17, CD4 T-cells, Tregs, SP-A and Caspase-3. Based on the immunopathophysiology, the selection of eight biomarkers in this study (sTREM-1, alveolar macrophage amount and function, IL-6, IL-17, CD4 T-cells, Tregs, SP-A and Caspase-3) could provide a better comprehensive in explaining the role of local innate and adaptive immune responses and its association with extubation failure and mortality. The presence of infectious disease can be marked by the presence of sTREM-1 [68]. Alveolar macrophage as a phagocyte plays a role in homeostasis, body protection, response to foreign particles, and tissue regeneration. Additionally, alveolar macrophage function regulates local immunology, especially lung immunity. The interaction of each cell will mediate the immune response. Alveolar macrophages will secrete cytokines and eicosanoid for neutrophil polymorphonuclear recruitment [69]. Kooguchi et al. [22] found that downregulation of alveolar macrophages will increase the severity of lung injury and mortality of pneumonia, and a study conducted by Puren et al. [23] found that a decrease/increase in cytokine (IL-6 and IL-17) levels are associated with severity of infection and inflammation. A study by Jedynak et al. [70] found that an increase in lung infection will stimulate sTREM-1 to activate monocyte and neutrophil and produce more inflammatory cytokines. Insufficient protection of alveolar macrophages to the infection will initiate adaptive immunity and activate lymphocytes (T cells), which then induce apoptosis (upregulated Caspase-3 and downregulated CD4 T-cells) [71]. Bielosludtseva et al. reported that reduction of T cell levels is associated with high mortality in pneumonia [30]. Additionally, a study by Shi et al. [38], reported that an increase in Caspase-3 is associated with increased alveolar epithelial apoptosis. Persistent severe lung injury will dysregulate lung epithelium regeneration (Tregs) and reduce surfactant production [61]. A study by Mukherjee et al. [36] reported that downregulated surfactant (SP-A) production will cause lung atelectasis and gas exchange disturbance. Previous studies explained that the reduction of immune cells and mediators will cause individual be more prone to diseases (especially infectious diseases) and/or will aggravate the existed disease condition [72].

This study reports significantly low levels of BALF CD4 T-cells count in extubation failure (*p* = .010) and non-survived (*p* = .018) critically ill COVID-19 pneumonia patients. Jiang et al. [8] reported a significantly low serum CD4 T-cells count in non-survived COVID-19 pneumonia patients. Vedder et al. [73] described normal BALF CD4 T-cells in ventilated COVID-19 pneumonia patients (BALF was collected within two days after mechanical ventilation), whereas in this study BALF was collected in an average of 4 h (SD = 0.82) after patients’ intubation. No significant differences were found in other BALF immunopathology biomarkers (sTREM-1, alveolar macrophage, IL-6, IL-17, Tregs, SP-A and Caspase-3) for both groups (extubation failure and mortality) in this study.

Some study limitations included: (1) the small number of study subjects may result in other biomarkers that were not statically significant. The lack of statistical significance in some BALF biomarkers may be explained by our current sample size being too small to detect the difference. Therefore, future studies with larger sample size are suggested to investigate the differences in other BALF biomarkers. (2) Chest CT scan imaging was not performed; hence, we could not assess other abnormalities that might be presented in the study patients. (3) No serial evolution time for BALF biomarkers to assess the dynamic changes.

## Conclusions

This study reports the association of low BALF CD4 T-cells count with early extubation failure and mortality in the severely affected lung of critically ill COVID-19 pneumonia patients. BALF CD4 T-cells count evaluation of severely affected lung can be an option to clinicians for assessment of early extubation failure and mortality in critically ill COVID-19 pneumonia patients.

## Data Availability

Our study data are available on request from the corresponding author (G.S.). The data are not publicly available due to their containing information that could compromise the privacy of study subjects.

## Appendix 1 BALF immunopathology biomarkers in critically ill COVID-19 pneumonia (less severely affected lung).

**Table:**

Biomarkers	Extubation success (*n* = 5)	Extubation failure (*n* = 23)	*p*
sTREM (ng/mg protein), median (IQR)	1.62 (1.09–2.00)	1.22 (0.84–2.28)	.697
Alveolar macrophage			
% (Macrophage cells/CD45+, HLA-DR+, CD11b+), median (IQR)	8.00 (5.10–9.10)	5.70 (2.70–10.80)	.472
MFI CD169, median (IQR)	598 (385–888)	628 (347.50–961.50)	.857
IL-6 (pg/mg protein), mean (±SD)	253.30 (±134.58)	393.04 (±315.57)	.346
IL-17 (pg/mg protein), median (IQR)	10.99 (10.58–11.08)	5.87 (4.54–8.56)	.051
CD4 (cell/μL), median (IQR)	37 (32–52)	23 (13.50–39)	.352
Foxp3+ CD25+/CD4 (%), median (IQR)	19.13 (13.86–19.23)	15.49 (12.24–33.18)	.697
Caspase 3 (ng/mg protein), median (IQR)	0.97 (0.57–1.05)	0.67 (0.36–1.26)	.881
SP-A (ng/mg protein), median (IQR)	13.03 (13.00–13.49)	12.56 (9.79–16.45)	.529

MFI: mean fluorescence intensity.

## Appendix 2 BALF immunopathology biomarkers in critically ill COVID-19 pneumonia (less severely affected lung).

**Table:**

Biomarkers	Survivors (*n* = 6)	Non-survivors (*n* = 22)	*p*
sTREM (ng/mg protein), median (IQR)	1.81 (1.09–2.09)	1.10 (0.81–2.42)	.502
Alveolar macrophage			
% (Macrophage cells/CD45+, HLA-DR+, CD11b+), median (IQR)	8.55 (5.10–10.90)	5.55 (2.60–10.70)	.275
MFI CD169, median (IQR)	491.50 (341–888)	649.50 (354–1007)	.538
IL-6 (pg/mg protein), median (IQR)	302.07 (145.26–425.74)	310.77 (121.75–575.43)	.780
IL-17 (pg/mg protein), median (IQR)	11.04 (10.58–12.58)	5.82 (4.28–8.00)	**.016**
CD4 (cell/μL), median (IQR)	34.50 (26–52)	21.50 (13–48)	.370
Foxp3+ CD25+/CD4 (%), median (IQR)	16.50 (13.84–19.28)	17.81 (10.97–37.91)	.576
Caspase 3 (ng/mg protein), median (IQR)	1.01 (0.57–1.07)	0.66 (0.33–1.09)	.370
SP-A (ng/mg protein), mean (±SD)	16.78 (±7.76)	13.01 (±5.24)	.170

MFI: mean fluorescence intensity.

## Appendix 3 Radiographic findings in critically ill COVID-19 pneumonia.

**Table:**

Chest X-ray	*n* = 28 (%)
Severely affected lung	
Right	19 (68)
Left	9 (32)
Baseline features	
Bilateral diffuse consolidation	27 (96)
Bilateral diffuse infiltrate	1 (3.5)
Pleural effusion	1 (3.5)
Pulmonary Nodules	2 (7)

## Abbreviations

ACE: angiotensin-converting enzyme
APACHE: acute physiology and chronic health evaluation
BAL: bronchoalveolar lavage
BALF: bronchoalveolar lavage fluid
CCI: Charlson Comorbidity Index
CD: cluster of differentiation
COVID-19: coronavirus disease 2019
CRP: C-reactive protein
ELISA: enzyme-linked immunoassay
ESR: erythrocyte sedimentation rate
ICU: intensive care unit
IFN: interferon
IL: interleukin
IQR: interquartile range
mSOFA: modified sequential organ failure assessment
PaO_2_/FiO_2_: the ratio of arterial oxygen partial pressure to fractional inspired oxygen
PBS: phosphate buffer saline
REU: resuscitation emergency unit
RT-PCR: real-time polymerase chain reaction
SARS: severe acute respiratory syndrome
SARS-CoV-2: severe acute respiratory syndrome coronavirus 2
SBT: spontaneous breathing trial
SD: standard deviation
SLE: systemic lupus erythematosus
SP-A: surfactant protein-A
sTREM-1: soluble triggering receptor expressed on myeloid cells-1
Tregs: regulatory T cells
TNF: tumour necrosis factor

## References

[1] Fernandez-Botran R, Uriarte SM, Arnold FW, et al. Contrasting inflammatory responses in severe and non-severe community-acquired pneumonia. Inflammation. 2014;37(4):1158–1166. 24557760 10.1007/s10753-014-9840-2 PMC7087758

[2] Coma E, Méndez-Boo L, Mora N, et al. Divergences on expected pneumonia cases during the COVID-19 epidemic in Catalonia: a time-series analysis of primary care electronic health records covering about 6 million people. BMC Infect Dis. 2021;21(1):283. 33740907 10.1186/s12879-021-05985-0 PMC7979451

[3] García LF. Immune response, inflammation, and the clinical spectrum of COVID-19. Front Immunol. 2020;11:1441. 32612615 10.3389/fimmu.2020.01441 PMC7308593

[4] Wendel Garcia PD, Fumeaux T, Guerci P, et al. Prognostic factors associated with mortality risk and disease progression in 639 critically ill patients with COVID-19 in Europe: initial report of the international RISC-19-ICU prospective observational cohort. EClinicalMedicine. 2020;25:100449. 32838231 10.1016/j.eclinm.2020.100449 PMC7338015

[5] Alharthy A, Aletreby W, Faqihi F, et al. Clinical characteristics and predictors of 28-day mortality in 352 critically ill patients with COVID-19: a retrospective study. J Epidemiol Glob Health. 2021;11(1):98–104. 33095982 10.2991/jegh.k.200928.001 PMC7958266

[6] Gamberini L, Tonetti T, Spadaro S, et al. Factors influencing liberation from mechanical ventilation in coronavirus disease 2019: multicenter observational study in fifteen Italian ICUs. J Intensive Care. 2020;8:80. 33078076 10.1186/s40560-020-00499-4 PMC7558552

[7] Ionescu F, Zimmer MS, Petrescu I, et al. Extubation failure in critically ill COVID-19 patients: risk factors and impact on in-hospital mortality. J Intensive Care Med. 2021;36(9):1018–1024. 34074160 10.1177/08850666211020281 PMC8173445

[8] Jiang Y, Abudurexiti S, An M-M, et al. Risk factors associated with 28-day all-cause mortality in older severe COVID-19 patients in Wuhan, China: a retrospective observational study. Sci Rep. 2020;10(1):22369. 33353956 10.1038/s41598-020-79508-3 PMC7755901

[9] Leoni MLG, Lombardelli L, Colombi D, et al. Prediction of 28-day mortality in critically ill patients with COVID-19: development and internal validation of a clinical prediction model. PLOS One. 2021;16(7):e0254550. 34255793 10.1371/journal.pone.0254550 PMC8277063

[10] Yu H, Luo J, Ni Y, et al. Early prediction of extubation failure in patients with severe pneumonia: a retrospective cohort study. Biosci Rep. 2020;40:BSR20192435. 31990295 10.1042/BSR20192435 PMC7007404

[11] Ciaccio M, Agnello L. Biochemical biomarkers alterations in coronavirus disease 2019 (COVID-19). Diagnosis. 2020;7(4):365–372. 32589600 10.1515/dx-2020-0057

[12] Raventós AA, Serpa Neto A. Biomarkers to guide ventilation management and readiness for extubation. Am J Respir Crit Care Med. 2021;203(10):1211–1212. 33503397 10.1164/rccm.202101-0093ED PMC8456467

[13] Paats MS, Bergen IM, Hanselaar WEJJ, et al. Local and systemic cytokine profiles in nonsevere and severe community-acquired pneumonia. Eur Respir J. 2013;41(6):1378–1385. 23258791 10.1183/09031936.00060112

[14] Ramirez P, Kot P, Marti V, et al. Diagnostic implications of soluble triggering receptor expressed on myeloid cells-1 in patients with acute respiratory distress syndrome and abdominal diseases: a preliminary observational study. Crit Care. 2011;15(1):R50. 21294874 10.1186/cc10015 PMC3221980

[15] Shi J-X, Li J-S, Hu R, et al. Diagnostic value of sTREM-1 in bronchoalveolar lavage fluid in ICU patients with bacterial lung infections: a bivariate meta-analysis. PLOS One. 2013;8(5):e65436. 23734253 10.1371/journal.pone.0065436 PMC3667178

[16] Al-Omari B, McMeekin P, Allen AJ, et al. Systematic review of studies investigating ventilator associated pneumonia diagnostics in intensive care. BMC Pulm Med. 2021;21(1):196. 34107929 10.1186/s12890-021-01560-0 PMC8189711

[17] Huh JW, Lim C-M, Koh Y, et al. Diagnostic utility of the soluble triggering receptor expressed on myeloid cells-1 in bronchoalveolar lavage fluid from patients with bilateral lung infiltrates. Crit Care. 2008;12(1):R6. 18205941 10.1186/cc6770 PMC2374623

[18] Gibot S, Cravoisy A, Levy B, et al. Soluble triggering receptor expressed on myeloid cells and the diagnosis of pneumonia. N Engl J Med. 2004;350(5):451–458. 14749453 10.1056/NEJMoa031544

[19] Guillon A, Arafa EI, Barker KA, et al. Pneumonia recovery reprograms the alveolar macrophage pool. JCI Insight. 2020;5(4):e133042. 10.1172/jci.insight.133042 PMC7101156 31990682

[20] Knapp S, Leemans JC, Florquin S, et al. Alveolar macrophages have a protective antiinflammatory role during murine pneumococcal pneumonia. Am J Respir Crit Care Med. 2003;167(2):171–179. 12406830 10.1164/rccm.200207-698OC

[21] Liao M, Liu Y, Yuan J, et al. Single-cell landscape of bronchoalveolar immune cells in patients with COVID-19. Nat Med. 2020;26(6):842–844. 32398875 10.1038/s41591-020-0901-9

[22] Kooguchi K, Hashimoto S, Kobayashi A, et al. Role of alveolar macrophages in initiation and regulation of inflammation in *Pseudomonas aeruginosa* pneumonia. Infect Immun. 1998;66(7):3164–3169. 9632581 10.1128/iai.66.7.3164-3169.1998 PMC108328

[23] Puren AJ, Feldman C, Savage N, et al. Patterns of cytokine expression in community-acquired pneumonia. Chest. 1995;107(5):1342–1349. 7750329 10.1378/chest.107.5.1342

[24] Montón C, Torres A, El-Ebiary M, et al. Cytokine expression in severe pneumonia: a bronchoalveolar lavage study. Crit Care Med. 1999;27:1745–1753. 10507593 10.1097/00003246-199909000-00008

[25] Wiedermann FJ, Lederer W. Inflammatory factors in alveolar lavage fluid from severe COVID-19 pneumonia: PCT and IL-6 in epithelial lining fluid. Open Med. 2021;16(1):1132–1133. 10.1515/med-2021-0330 PMC8354377 34435137

[26] Wonnenberg B, Jungnickel C, Honecker A, et al. IL-17A attracts inflammatory cells in murine lung infection with *P. aeruginosa*. Innate Immun. 2016;22(8):620–625. 27634821 10.1177/1753425916668244

[27] Ritchie ND, Ritchie R, Bayes HK, et al. IL-17 can be protective or deleterious in murine pneumococcal pneumonia. PLoS Pathog. 2018;14(5):e1007099. 29813133 10.1371/journal.ppat.1007099 PMC5993294

[28] Orlov M, Dmyterko V, Wurfel MM, et al. Th17 cells are associated with protection from ventilator associated pneumonia. PLOS One. 2017;12(8):e0182966. 28806403 10.1371/journal.pone.0182966 PMC5555641

[29] Tsai HC, Velichko S, Hung LY, et al. IL-17A and Th17 cells in lung inflammation: an update on the role of Th17 cell differentiation and IL-17R signaling in host defense against infection. Clin Dev Immunol. 2013;2013:267971. 23956759 10.1155/2013/267971 PMC3730142

[30] Bielosludtseva K, Pertseva T, Kyreeva T. CD4 as a predictor of systemic inflammatory response and death at severe community-acquired pneumonia. Eur Respir J. 2013;42:4366.

[31] Jambo KC, Sepako E, Fullerton DG, et al. Bronchoalveolar CD4+ T cell responses to respiratory antigens are impaired in HIV-infected adults. Thorax. 2011;66(5):375–382. 21357587 10.1136/thx.2010.153825 PMC3088469

[32] Park H, Li Z, Yang XO, et al. A distinct lineage of CD4 T cells regulates tissue inflammation by producing interleukin 17. Nat Immunol. 2005;6(11):1133–1141. 16200068 10.1038/ni1261 PMC1618871

[33] Neill DR, Fernandes VE, Wisby L, et al. T regulatory cells control susceptibility to invasive pneumococcal pneumonia in mice. PLoS Pathog. 2012;8(4):e1002660. 22563306 10.1371/journal.ppat.1002660 PMC3334885

[34] Wang Y, Zheng J, Islam MS, et al. The role of CD4(+)FoxP3(+) regulatory T cells in the immunopathogenesis of COVID-19: implications for treatment. Int J Biol Sci. 2021;17(6):1507–1520. 33907514 10.7150/ijbs.59534 PMC8071774

[35] Adamzik M, Broll J, Steinmann J, et al. An increased alveolar CD4 + CD25 + Foxp3 + T-regulatory cell ratio in acute respiratory distress syndrome is associated with increased 30-day mortality. Intensive Care Med. 2013;39(10):1743–1751. 23949701 10.1007/s00134-013-3036-3 PMC7095258

[36] Mukherjee S, Giamberardino C, Thomas JM, et al. Surfactant protein a modulates induction of regulatory T cells via TGF-β. J Immunol. 2012;188(9):4376–4384. 22474025 10.4049/jimmunol.1101775 PMC3331948

[37] Du X, Meng Q, Sharif A, et al. Surfactant proteins SP-A and SP-D ameliorate pneumonia severity and intestinal injury in a murine model of *Staphylococcus aureus* pneumonia. Shock. 2016;46(2):164–172. 26849628 10.1097/SHK.0000000000000587

[38] Shi S, Liu X, Li H. Downregulation of caspase-3 alleviates Mycoplasma pneumoniae-induced apoptosis in alveolar epithelial cells. Mol Med Rep. 2017;16(6):9601–9606. 29039549 10.3892/mmr.2017.7782

[39] Kosai K, Seki M, Tanaka A, et al. Increase of apoptosis in a murine model for severe pneumococcal pneumonia during influenza a virus infection. Jpn J Infect Dis. 2011;64(6):451–457. 22116322

[40] Baughman RP, Sternberg RI, Hull W, et al. Decreased surfactant protein A in patients with bacterial pneumonia. Am Rev Respir Dis. 1993;147(3):653–657. 8442601 10.1164/ajrccm/147.3.653

[41] Mandell LA, Wunderink RG, Anzueto A, et al. Infectious Diseases Society of America/American Thoracic Society Consensus Guidelines on the management of community-acquired pneumonia in adults. Clin Infect Dis. 2007;44(Suppl. 2):S27–S72. 17278083 10.1086/511159 PMC7107997

[42] Feng F, Jiang Y, Yuan M, et al. Association of radiologic findings with mortality in patients with avian influenza H7N9 pneumonia. PLOS One. 2014;9(4):e93885. 24705783 10.1371/journal.pone.0093885 PMC3976364

[43] Hunninghake GW, Gadek J, Kawanami O, et al. Inflammatory and immune processes in the human lung in health and disease: evaluation by bronchoalveolar lavage. Am J Pathol. 1979;97(1):149–206. 495693 PMC2042387

[44] Baldwin DR, Wise R, Andrews JM, et al. Microlavage: a technique for determining the volume of epithelial lining fluid. Thorax. 1991;46(9):658–662. 1948795 10.1136/thx.46.9.658 PMC463361

[45] Nayak DK, Mendez O, Bowen S, et al. Isolation and in vitro culture of murine and human alveolar macrophages. J Vis Exp. 2018. 10.3791/57287 PMC6100701 29733312

[46] Saris A, Reijnders TDY, Nossent EJ, et al. Distinct cellular immune profiles in the airways and blood of critically ill patients with COVID-19. Thorax. 2021;76(10):1010–1019. 33846275 10.1136/thoraxjnl-2020-216256 PMC8050882

[47] Baron A, Hachem M, Tran Van Nhieu J, et al. Bronchoalveolar lavage in patients with COVID-19 with invasive mechanical ventilation for acute respiratory distress syndrome. Ann Am Thorac Soc. 2021;18(4):723–726. 33233944 10.1513/AnnalsATS.202007-868RL PMC8009009

[48] Dentone C, Vena A, Loconte M, et al. Bronchoalveolar lavage fluid characteristics and outcomes of invasively mechanically ventilated patients with COVID-19 pneumonia in Genoa, Italy. BMC Infect Dis. 2021;21(1):353. 33858331 10.1186/s12879-021-06015-9 PMC8049078

[49] Gelarden I, Nguyen J, Gao J, et al. Comprehensive evaluation of bronchoalveolar lavage from patients with severe COVID-19 and correlation with clinical outcomes. Hum Pathol. 2021;113:92–103. 33905777 10.1016/j.humpath.2021.04.010 PMC8159767

[50] Voiriot G, Fajac A, Lopinto J, et al. Bronchoalveolar lavage findings in severe COVID-19 pneumonia. Intern Emerg Med. 2020;15(7):1333–1334. 32415560 10.1007/s11739-020-02356-6 PMC7225401

[51] Yao Y, Cao J, Wang Q, et al. D-dimer as a biomarker for disease severity and mortality in COVID-19 patients: a case control study. J Intensive Care. 2020;8:49. 32665858 10.1186/s40560-020-00466-z PMC7348129

[52] Cerda-Mancillas MC, Santiago-Germán D, Andrade-Bravo B, et al. D-Dimer as a biomarker of severity and adverse outcomes in patients with community acquired pneumonia. Arch Med Res. 2020;51(5):429–435. 32402575 10.1016/j.arcmed.2020.04.014

[53] King CS, Sahjwani D, Brown AW, et al. Outcomes of mechanically ventilated patients with COVID-19 associated respiratory failure. PLOS One. 2020;15(11):e0242651. 33227024 10.1371/journal.pone.0242651 PMC7682899

[54] Wong HYF, Lam HYS, Fong AH-T, et al. Frequency and distribution of chest radiographic findings in patients positive for COVID-19. Radiology. 2020;296(2):E72–E78. 32216717 10.1148/radiol.2020201160 PMC7233401

[55] Yasin R, Gouda W. Chest X-ray findings monitoring COVID-19 disease course and severity. Egypt J Radiol Nucl Med. 2020;51(1):193.

[56] Sinha V, Chhaya V, Barot DS, et al. Foreign body in tracheobronchial tree. Indian J Otolaryngol Head Neck Surg. 2010;62(2):168–170. 23120706 10.1007/s12070-010-0044-2 PMC3450294

[57] Huffnagle GB, Dickson RP. The bacterial microbiota in inflammatory lung diseases. Clin Immunol. 2015;159(2):177–182. 26122174 10.1016/j.clim.2015.05.022 PMC5081074

[58] Chaudhry R, Bordoni B. StatPearls. StatPearls Publishing; 2021.

[59] Martinez-Sanchez ME, Huerta L, Alvarez-Buylla ER, et al. Role of cytokine combinations on CD4+ T cell differentiation, partial polarization, and plasticity: continuous network modeling approach. Front Physiol. 2018;9:877. 30127748 10.3389/fphys.2018.00877 PMC6089340

[60] Corthay A. How do regulatory T cells work? Scand J Immunol. 2009;70(4):326–336. 19751267 10.1111/j.1365-3083.2009.02308.x PMC2784904

[61] Mock JR, Garibaldi BT, Aggarwal NR, et al. Foxp3+ regulatory T cells promote lung epithelial proliferation. Mucosal Immunol. 2014;7(6):1440–1451. 24850425 10.1038/mi.2014.33 PMC4205163

[62] Norton DL, Ceppe A, Tune MK, et al. Bronchoalveolar Tregs are associated with duration of mechanical ventilation in acute respiratory distress syndrome. J Transl Med. 2020;18(1):427. 33176790 10.1186/s12967-020-02595-3 PMC7656499

[63] Burns GD, Phillips J, Pangilinan LP, et al. Time to extubation with and without COVID-19 pneumonia. Respir Care. 2021;66:3607976.

[64] Funk G-C, Anders S, Breyer M-K, et al. Incidence and outcome of weaning from mechanical ventilation according to new categories. Eur Respir J. 2010;35(1):88–94. 19541716 10.1183/09031936.00056909

[65] Davidson KR, Ha DM, Schwarz MI, et al. Bronchoalveolar lavage as a diagnostic procedure: a review of known cellular and molecular findings in various lung diseases. J Thorac Dis. 2020;12(9):4991–5019. 33145073 10.21037/jtd-20-651 PMC7578496

[66] Zeng F, Huang Y, Guo Y, et al. Association of inflammatory markers with the severity of COVID-19: a meta-analysis. Int J Infect Dis. 2020;96:467–474. 32425643 10.1016/j.ijid.2020.05.055 PMC7233226

[67] Pandolfi L, Fossali T, Frangipane V, et al. Broncho-alveolar inflammation in COVID-19 patients: a correlation with clinical outcome. BMC Pulm Med. 2020;20(1):301. 33198751 10.1186/s12890-020-01343-z PMC7668012

[68] Cao C, Gu J, Zhang J. Soluble triggering receptor expressed on myeloid cell-1 (sTREM-1): a potential biomarker for the diagnosis of infectious diseases. Front Med. 2017;11(2):169–177. 28425045 10.1007/s11684-017-0505-z

[69] Gordon SB, Read RC. Macrophage defences against respiratory tract infections. Br Med Bull. 2002;61:45–61. 11997298 10.1093/bmb/61.1.45

[70] Jedynak M, Siemiatkowski A, Mroczko B, et al. Soluble TREM-1 serum level can early predict mortality of patients with sepsis, severe sepsis and septic shock. Arch Immunol Ther Exp. 2018;66(4):299–306. 10.1007/s00005-017-0499-x PMC6061141 29282483

[71] Kemp K, Bruunsgaard H, Skinhøj P, et al. Pneumococcal infections in humans are associated with increased apoptosis and trafficking of type 1 cytokine-producing T cells. Infect Immun. 2002;70(9):5019–5025. 12183548 10.1128/IAI.70.9.5019-5025.2002 PMC128234

[72] Reynolds JH, McDonald G, Alton H, et al. Pneumonia in the immunocompetent patient. Br J Radiol. 2010;83(996):998–1009. 21088086 10.1259/bjr/31200593 PMC3473604

[73] Vedder V, Schildgen V, Lüsebrink J, et al. Differential cytology profiles in bronchoalveolar lavage (BAL) in COVID-19 patients: a descriptive observation and comparison with other corona viruses, influenza virus, *Haemophilus influenzae*, and *Pneumocystis jirovecii*. Medicine. 2021;100(1):e24256. 33429831 10.1097/MD.0000000000024256 PMC7793419

